# No relationship between most polymorphisms of steroidogenic acute regulatory (*StAR*) gene with polycystic ovarian syndrome

**Published:** 2015-12

**Authors:** Azadeh-Sadat Nazouri, Mona Khosravifar, Ali-Asghar Akhlaghi, Marzieh Shiva, Parvaneh Afsharian

**Affiliations:** 1 *Department of Biology, Sciences Faculty, Shahid Beheshti University, Tehran, Iran.*; 2 *Department of Genetics, Reproductive Biomedicine Research Center, Royan Institute for Reproductive Biomedicine, ACECR, Tehran, Iran.*; 3 *Department of Epidemiology and Reproductive Health, Reproductive Epidemiology Research Center, Royan Institute for Reproductive Biomedicine, ACECR, Tehran, Iran.*; 4 *Department of Endocrinology and Female Infertility, Reproductive Biomedicine Research Center, Royan Institute for Reproductive Biomedicine, ACECR, Tehran, Iran.*

**Keywords:** * Polycystic ovary syndrome*, *Steroidogenic acute regulatory (StAR) gene*, *Single nucleotide polymorphism*

## Abstract

**Background:**

Polycystic ovary syndrome (PCOS) is one of the most common endocrine women’s disorders in reproductive age. Hyperandrogenism has a critical role in the etiology of PCOS and it can cause fault in Steroidogenesis process. During steroidogenesis, steroidogenic acute regulatory protein (*StAR*) seems to increase the delivery of cholesterol through mitochondrial membrane. Therefore, polymorphisms of *StAR* might effect on this protein and play a role in the etiology of PCOS.

**Objective:**

The aim of this study was to investigate the association between *StAR* SNPs with PCOS. Thus, seven polymorphisms in this gene: rs104894086, rs104894089, rs104894090, rs137852689, rs10489487, rs104894085 were detected.

**Materials and Methods:**

In this case control study, 45 PCOS women, 40 male factor/unexplained infertile women, and 40 fertile women as two control groups were participated from 2008-2012. Polymorphisms were detected using restriction fragment length polymorphism (PCR-RFLP) method.

**Results:**

Heterozygote genotyping for rs137852689 SNP (amino acid 218 C > T) was only seen in seven PCOS patients, one in normal ovulatory women, and five in male factor/unexplained infertile women (15.5%, 2.5%, 12.5%, respectively) (p= 0.12). While, it has shown no association between other SNPS with PCOs.

**Conclusion:**

The RFLP results for seven chosen SNPs, which located in exon 5 and 7 showed normal status in three groups, it means no heterozygous or homozygous forms of selected SNPs were observed. So, it seems evaluation of the active amino acid sites should be investigated and also the study population should be increased.

## Introduction

Polycystic ovary syndrome (PCOS) is the most common women’s disorder that according to the latest reports of the PCOS Consensus Workshop Group, is affecting 6-10% of the population and according to the National Institutes of Health (NIH) criteria, affects 15% of reproductive aged women (1). It also causes 91% of anovulatory infertility in women (2). It is characterized by hyper-androgenism, oligomenorrhea or amenorrhea, anovulatory cycle and presence of multiple cysts of ovaries on the ultrasound examination. On the other hand, obesity and high levels of luteinizing hormone (LH) and insulin resistance are other symptoms of this disease (3-5). Restoring ovulation is the aim of PCOS treatment. In this regard, drugs used in PCOS treatment is aromatase inhibitors (AIs) like letrozol, which is co-administered with gonodotrapins to improve ovulation and clomiphene citrate (CC), which is the first treatment to stimulate ovulation of PCOS patients who are undergoing Intrauterine Insemination (IUI). In the second stage of treatment, recombinant Follicle-stimulating hormone (rFSH) and metformin as insulin sensitizers are prescribed. Different diagnosis of PCOS markers and also different responses to treatment because of individual pharmacogenetic aspects, make PCOS a heterogeneous syndrome (3).

As mentioned above, patients with PCOS have an abnormality in androgen biosynthesis resulting in hyperandrogenemia (3, 6). The first step in androgen biosynthesis is transporting cholesterol through the *StAR* protein from outer to the inner mitochondrial membrane (7, 8). Gonads, placenta, adrenal glands and central nervous system are places that steroid hormones are synthesized. Regardless of the places that steroid hormones synthetize, a common precursor of all steroid hormones is cholesterol. The delivery of cellular cholesterol from the outer to the inner mitochondrial membrane, where the cytochrome p450 side chain cleavage enzyme exists, is the first and rate limiting step of steroidogenesis (9). Several proteins are candidates for the delivery of cholesterol, but *StAR* protein is the best candidate to do this role (7, 9). Pol *et al* discovered a mitochondrial protein classified as phosphoproteins, which had 30KDa weight (10), and it was purified, sequenced and expressed in 1994 as a new protein named *StAR* (9,11). 

*StAR* protein has 284 amino acids (12) and it has been cloned and highly conserved in many species such as mammals, birds, amphibians, and fish (8). Human adrenal cortex, ovary, testis and kidney were tissues that *StAR* mRNA has been found in them and steroidogenic factor 1, GATA-4, insulin-like growth factor (IGF) -I and GATA enhancer binding protein have been known as regulators of *StAR* protein (7). *StAR* gene is located on the short arm of chromosome 8 in the region 8p11.2. Its pseudo gene is located on chromosome 13 (13) with the length of 8 kbp, containing 7 exons and 6 introns (14). The N- terminal with 62 amino acids as a mitochondrial targeting sequence and a site that transfers cholesterol across the mitochondrial membrane as the *StART* domain consisting of 210 amino acids are mainly two functional domains of *StAR *protein (11, 12).

Since *StAR* has a critical role in binding to cholesterol, its facility to transfer it stimulates and initiates the process of steroidogenesis (6, 12). On the other hand, findings indicated that PCOS patients have high levels of steroid hormones so *StAR* may result in the abnormality of steroidogenesis found in the PCOS patients. Investigations indicated that variations located between exons 5 and 7 or affected the *StAR*- related lipid transfer (*StART*) domain were the most mutations that are seen in *StAR* gene (11).

Therefore in this study, the most significant single nucleotide polymorphisms (SNPs) of this gene (Table I), were investigated in PCOS patients who were undergone IUI treatment.

## Materials and methods

In this case control study, 125 Iranian women from 2008-2012 referred to Royan Institiute, Tehran, Iran, in three groups were included group 1) women with PCOS (n= 45), group 2) healthy women (n= 40) who had at least one child with normal fertilization that have been chosen from healthy volunteered females, and group 3) male factor/unexplained infertile women (n= 40) as a drug response control group in order to evaluate the association of chosen SNPs with drug response in comparison with PCOS patients.

Women in groups 1 and 3 have been undergone intrauterine insemination (IUI) during 2008-2012 at Royan Infertility Institute, and were administered clomiphene citrate/letrozole, 100 mg/day from the days three to seven of their menstrual cycle according to the ovulation induction protocol and were prescribed gonadotropin according to their ovarian response.

PCOS women with a history of ovary cutter, using other medication in past 3 months, and older than 35 years were excluded from the study. Our study was approved by the Ethics Committee of Royan Institute, Tehran, Iran, and all cases signed an informed consent.

**Clinical data**

Clinical data, such as body mass index (BMI), age, and follicles’ number and diameter in 10^th^ day of menstruation and therapy cycles were collected from the patient’s records.

The comparison was done between two groups (groups 1 and 3) and group 2 which was chosen just for detecting. The SNPs was omitted in this comparison. To study the similarities between these two groups (1 and 3), affecting and potentially confounding factors was investigated. It is worth mentioning that these variables were not confounded (Table II (.

**SNPs and polymorphism genotyping analysis**

Selected polymorphisms were chosen from the NCBI site (http://www.ncbi.nlm.nih.gov/clinicalsnp/) (14) and based on gene alternation of *StAR* deficiency disorders (Table I).

Genomic DNA samples were collected from the Royan DNA Bank, which had been isolated from peripheral blood by salting out method. Primer pairs were designed for each of the three studied exons (Table III).

Primer 3 software (http://primer3.ut.ee/) was used to design all primers, that were set in specific temperatures. Genotyping of 7 chosen polymorphisms of *StAR* gene were studied by Polymerase Chain Reaction-Restriction Fragment Length Polymorphism (PCR-RFLP).

**Figure1:**
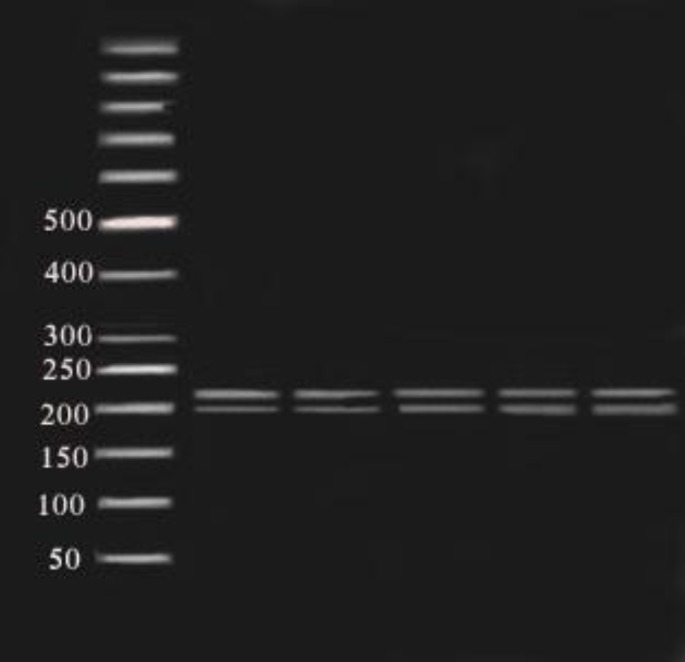
Fragments (181,210 bp) of NmuCI enzyme digestion of amplified exon 5 (391bp) on 2% agarose gel in 80 constant voltage in RFLP method. 50 bp ladder was used.

**Figure 2:**
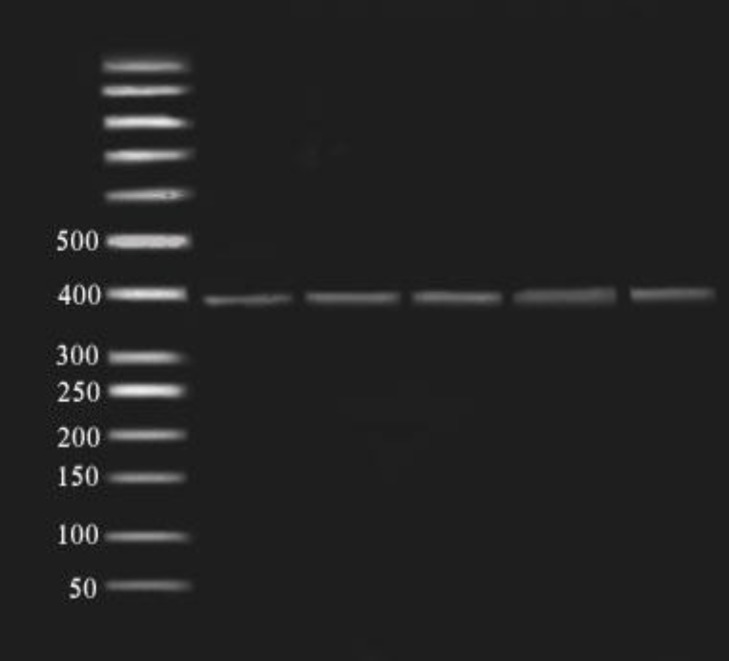
Fragments (391bp) of SphI enzyme digestion of amplified exon 5 (391bp) on 2% agarose gel in 80 constant voltage in RFLP method. 50 bp ladder was used

**Figure 3:**
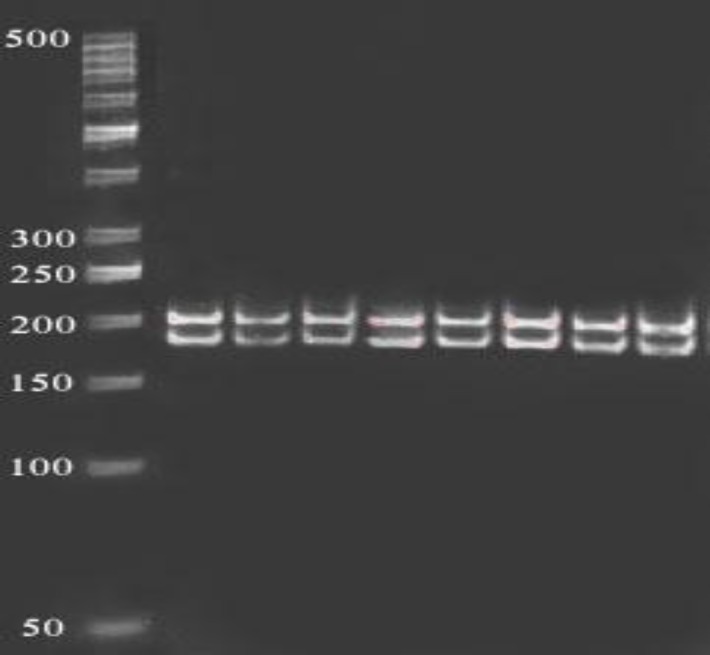
Fragments (12, 181, 198 bp) of HinP1I enzyme digestion of amplified exon 5 (391 bp) on 2% polyacrylamide gel in 80 constant voltage in RFLP method. 50 bp ladder was used (polyacrylamide gel was used to identify more exactly between fragments

**Figure 4:**
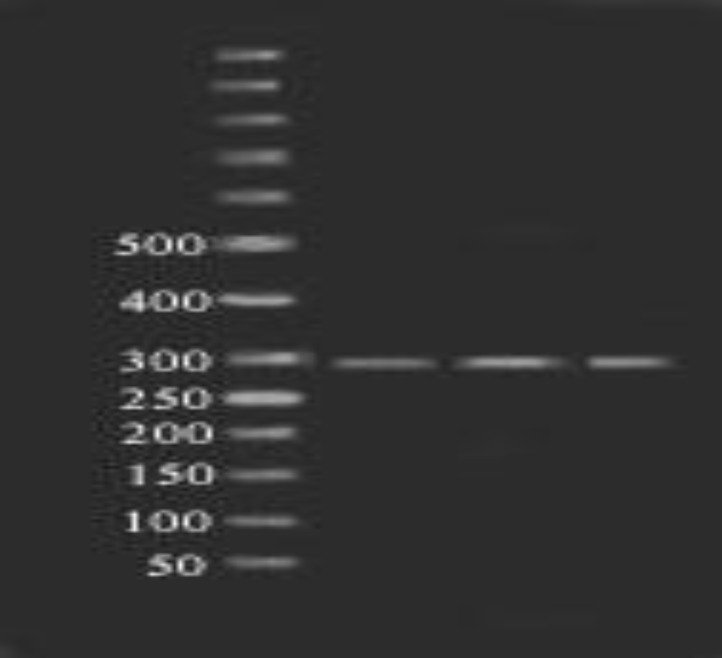
Fragments (299 bp) of BtgI enzyme digestion of amplified exon 6 (299bp) on 2% agarose gel in 80 constant voltage in RFLP method. 50 bp ladder was used

**Figure 5:**
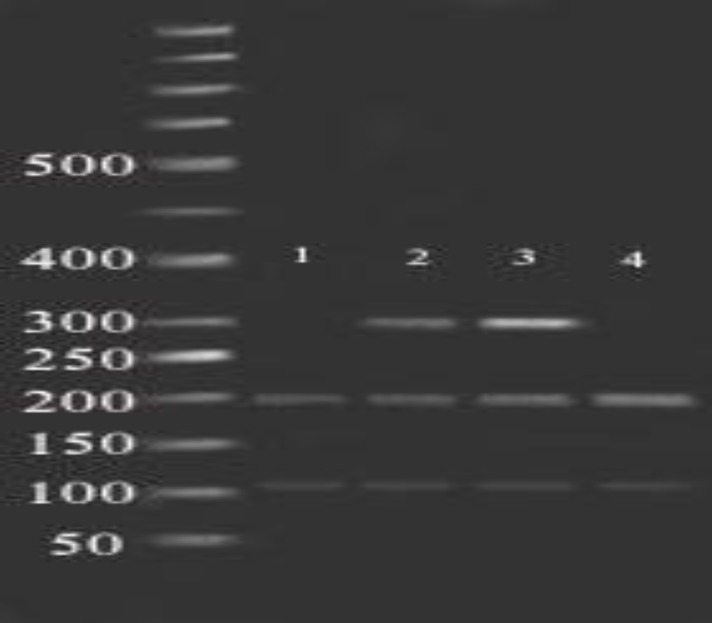
Fragments (101, 198 bp) of Ecil enzyme digestion of amplified exon 6 (299bp) on 2% agarose gel in 80 constant voltage in RFLP method. Lane 1 and 4 contain wild type forms and lane 2 and 3 are the heterozygote of SNP rs137852689. 50 bp ladder was used

**Figure 6:**
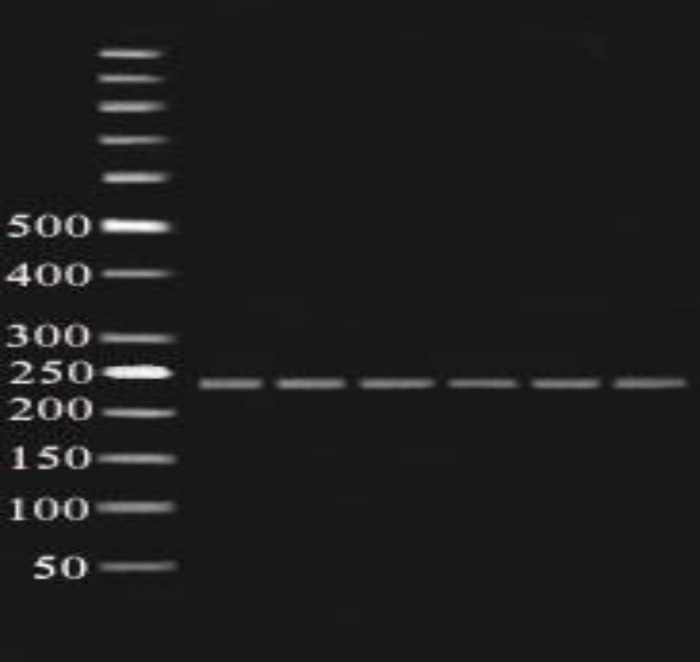
Fragments (48, 227bp) of AIuI enzyme digestion of amplified exon 7 (275bp) on 2% agarose gel in 80 constant voltage in RFLP method. 50 bp ladder was used (48 bp band had run out of the gel

**Figure 7:**
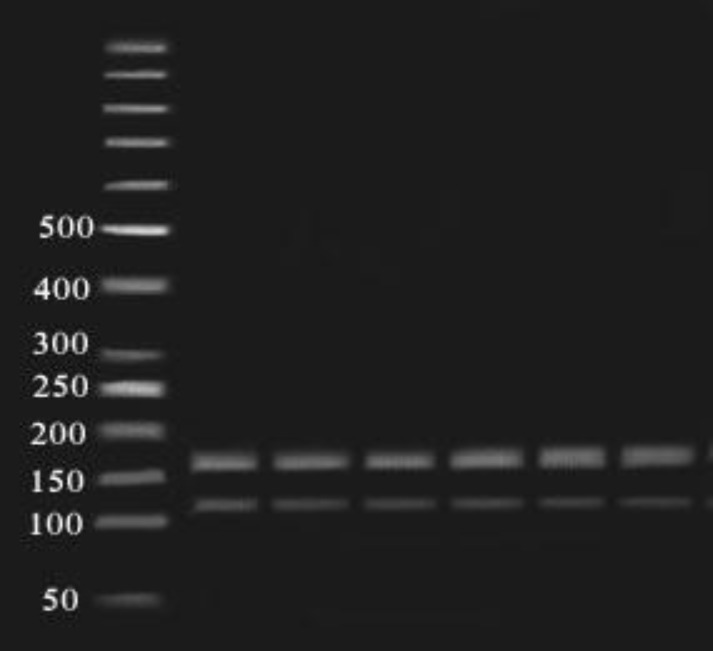
Fragments (115, 160 bp) of AlwNI enzyme digestion of amplified exon 7 (275bp) on 2% agarose gel in 80 constant voltage in RFLP method. 50 bp ladder was used

Polymerase chain reaction (PCR) was used to amplify genomic DNA in a 50 μl reaction volume containing 30 ng genomic DNA, 1-1.5Mm MgCl2, 0.2 Mm dNTP, 0.4 pMol of each primer and 0.6 unit/μl Tag DNA polymerase enzyme (Cinnagene) in the 1X PCR buffer. For exon 6, 25μl Ampliqon Tag DNA polymerase, 2X master mix with 1.5 Mm MgCl2 were used. The PCR was performed using Eppendorf gradient thermal cycler as follows: an initial denaturation at 94^°^C for 4 min, followed by 35 cycles of denaturation at 94^°^C for 30 seconds, annealing at a defined temperature (Table III) for 30 seconds, and extension stage at 72^°^C for 60 seconds with a final extension at 72^°^C for 10 min. All PCR products were visualized under UV light using ethidium bromide staining by 1.2-2% agarose gel electrophoresis. Related restriction enzyme (RFLP) was done on PCR products to identify SNPs. Seven enzymes were used. NmuCI, SphI and HinP1I for exon 5 (figures1-3, respectively), AIuI and AIwNI for exon 7 (figure 6 and 7, respectively) all were purchased from Fermentase fastdigest. 8 μl for exon 5 and 10 μl for exon 7 PCR products were incubated with one unit of each determined enzyme in 37^°^C at least for 5 minutes separately. For exon 6 EciI and BtgI (figure 4 and 5 respectively) enzymes were provided from New England Biolab (England). 1 unit EciI and 3 units BtgI enzyme were needed to digest 12 μl PCR products in 37^°^C during 1 hour. The fragments were separated by 2% agarose gel electrophoresis and visualized by ethidium bromide staining. The size of fragments achieved has been shown in table IV.

**Statistical analysis**

Mean ± SEM (SD) and frequency (proportion) were used to describe cases and controls quantitative and qualitative clinical characteristics, respectively. For quantitative treatment response (diameter and number of follicles) independent sample *t* test was used to determine the significant difference between group 1 and 3. Group 2 was excluded from this comparison of the diameter and number of follicules. For investigating the potential confounding variables in the comparison of group1 and 3, all baseline characteristics were compared between these two groups using t-test or Chi-square test where appropriated. All statistical analysis was performed using Statistical Package for the Social Sciences, version 16.0, SPSS Inc., Chicago, Illinois, USA (SPSS software) and p-value ≤ 0.05 were considered statistically significant.

For treatment response (follicular diameter) Chi-square and independent sample *t-*test were used. 

## Results

**Clinical data**

The demographic, clinical and endocrinological characteristics of participants are showed in table II. There were no significant differences in mean age and BMI between two groups (Table II).

**Pharmacogenetic characteristics**

The results of consuming clomiphene citrate in group 1 and group 3 (two treated groups) showed that 64% of group 1 and 35% of group 3 had resistance to this drug, which showed a statistically significant difference between them in usage of Clomiphene Citrate and Letrozol (p= 0.01). On the other hand, data have suggested that no significant difference was seen between the effect of Letrozol and Clomiphene Citrate on treatment response, according to the two parameters; number (p=0.86, p=0.76, respectively) and diameter of follicles (p=0.73, p= 0.30) on the 10^th^ day of menstruation.

**Genotype analysis**

The RFLP results for the SNPs located in exon 5 and 7 indicated that these polymorphisms were in normal status in three groups, which means no heterozygous or homozygous forms of SNPs were observed. For the polymorphism Arg 217 Thr (located in exon 6), no heterozygous form was seen. The digested fragments of PCR products using Btg enzyme showed a normal length on the electrophoresis gel for this SNP (Table IV) and there was no association between PCOS treatment and this polymorphism (p=0.12). EciI enzyme was used for genotyping Ala 218 Val polymorphism (rs137852689) located in exon 6. 

A number of the samples in all three groups indicated the heterozygote form of this SNP (seven in group 1, one in group 2, and five in group 3). 

**Figure 8:**
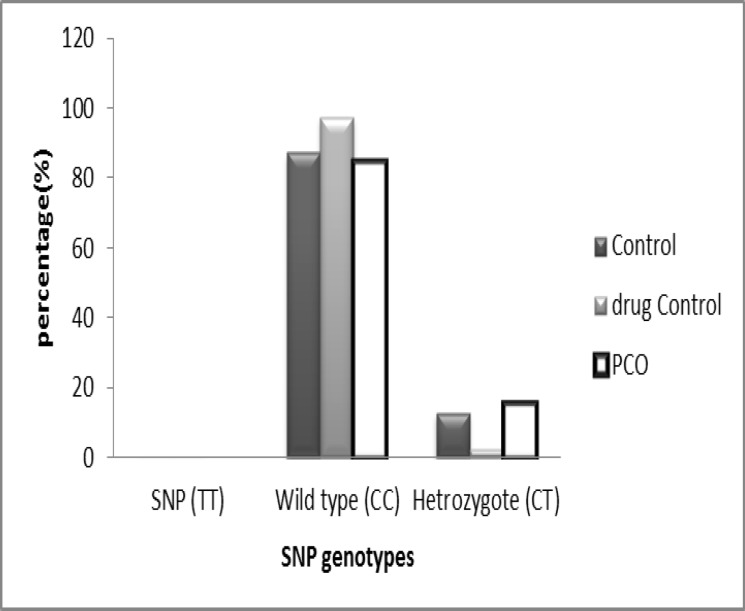
Genotype frequency of rs137852689 polymorphism (C218T) in *StAR* gene for the three studied groups (p= 0.12).

It must be mentioned that no homozygocity of this SNP was seen. Although the genotype distributions of SNP rs137852689 (CTT) versing wild type (CCT) in PCOS patients (7% vs. 93.4%) were different from normal ovulatory women (2.5% vs. 97.5%) and fertile control group (12.5 % vs. 87.5%), no significant association was seen (p=0.12) (Figure 8). 

Allele frequencies were observed as follows: the normal allele (C) was 92% in 45 PCOS patients, 93% in 40 fertile control subjects and 98% in 40 normal ovulatory women, and the SNP allele (T) was 8%, 7%, and 2%, respectively.

**Table I:** Location and details of *StAR* SNPs

**Polymorphism ref NO.**	**Nucleotide change**	**Amino acid change**	**Exon**
rs104894086	G/T G/A	Arg 182 His Arg 182 Lue	5
rs104894089	G/A	Met 187 Val	5
rs104894090	C/T	Cys 188 Arg	5
----------------	G/C	Arg 217 Thr	6
rs137852689	C/T	Ala 218 Val	6
rs10489487	G/A	Trp 250 Ter	7
rs104894085	C/T	Gln 258 Ter	7

**Table II:** Demographic, clinical and endocrinological characteristics of participants in group 1and 3

**Variable**	**Group 1 (n= 45)**	**Group 3 (n= 40)**	**p-value**
Body weight (Kg) *	68.21±14.8	64±12	0.95
Body mass index (Kg/m^2^ ) *	26.82±3.8	25.12±4.3	0.97
Age (year) *	27.25±4.0	27.72 ±3.0	0.19
Basal LH levels (mIU/mL) *	9.38±6.0	5.45±4.7	0.99
Basal FSH levels (mIU/mL) *	6.22 ±3.6	5.56±2.1	0.78
LH/FSH ratio*	1.63±0. 9	1.01±0.7	0.99
Follicle number ≥15 mm,on day 10*(n)	1.99±1.8	2.47±1.7	0.10
Ovulation rate (%)	19.8	24.7	0.61
Medication (Clomiphene; Letrozol) (%)	38.3; 61.7	63.6; 36.4	0.016

LH: luteinizing hormone; FSH: Follicle-stimulating hormone.

Independent *t *test was used for body weight, body mass index, Aae, basal LH levels, basal FSH levels, and LH/FSH ratio.

Chi-Square test was used for follicle number ≥ 15 mm, on day 10, ovulation rate, and medication.

* Data are presented as mean ± standard deviation (SD).

**Table III:** Information of designed primers

**Exon**	Primer sequence (5′-3′)	**TM (** ^°^ **C)**	**Amplication size (bp)**
5	F: AGCCCAGTGTGAATGCTGTA R: CAAGGGTTGGTTTCTTGGAG	57.6	391
6	F: GTGTTATCTATGGTACTGGTGT R:TCTTACAGCCTGTGATTCTATCAG	63	299
7	F: GCAGCCTGTTTGTGATAGGG R: GCAGGCTTCCAGTAGGGATT	59.7	275

F, forward primer,

R, reverse primer,

Tm, annealing temperature,

bp, base pairs.

**Table IV:** Restriction enzymes for RFLP analysis of *StAR* polymorphisms

**Enzyme name**	**SNP position**	**Digestion conditions**	**RFLP fragments in SNP (bp)**	**Amplication size (bp)**	**FLP fragments in normal alleles (bp)**
NmuCI	Exon5/ aa182	5 min/ 37° C	391	391	210/181
SphI	Exon5/ aa187	5 min/ 37° C	193/198	391	391
HinP1I	Exon5/ aa188	5 min/ 37° C	181/210	391	181/12/198
BtgI	Exon6/ aa217	1 hour/37°C	81/218	299	299
EciI	Exon6/ aa218	1 hour/ 37° C	299	299	198/101
AIuI	Exon7/ aa250	5 min/ 37° C	48/139/88	275	48/227
AlwNI	Exon7/ aa250	5 min/ 37° C	275	275	160/115

RFLP: Restriction fragment length polymorphism

## Discussion

The RFLP results for our seven chosen SNPs were showed, normal status in three mentioned groups, it means on heterozygous or homozygous forms were observed in this study. The PCOS is a common hormonal disorder with changing in the balance of steroids (15). 

When LH, according to the ”two cells- two gonadotropins theory” affects it’s receptors on theca cells, the transported cholesterol, which is the precursor of all steroids hormones convert to androgen (3). The mechanism of cholesterol transport is important for steriodogenesis. 

In this regard, since 40 years ago, previous studies had showed that steroidogenesis blockage can be caused by inhibitors of protein synthesis. More studies have shown that this blockage of steroidogenesis occurred in early steps of steroidogenesis, especially in the formation of pergnenolone, which means these inhibitors prevented the movement of cholesterol from the outer to inner mitochondrial membrane (11). On the other hand, when adrenal cells were stimulated with cAMP, synthesis of a family of 30-37KDa proteins was the first detectable event (16). 

Strauss *et al* stated that the cultures of PCOS theca cells produced greater amount of testosterone than normal theca cells and suggested that regulation of steriodogenesis in PCOS theca cells shows genetic abnormalities (17). 

Since, *StAR *protein has a critical role in facilitating of steroidogenesis (18); it can be a suitable candidate for hormonal abnormalities in PCOS. According to this, in 2005 Jakubowski included *StAR* as one of the candidate genes involved in PCOS (19). 

So, any mutation in *StAR* protein, especially in cholesterol binding sites, causes hormonal abnormalities such as Lipoid Congenital Adrenal Hyperplasia, PCOS (13), and endometriosis (20). 

In 2001, Kashar-Miller *et al* stated that changes in *StAR* gene can cause PCOS as a reason in the earliest steps of androgen biosynthesis. They examined *StAR* protein in ovaries of PCOS patients and healthy women by Western blotting (WB) (six samples in each group) and immunohistochemistry (IHC) (seven and ten samples, respectively). Although, they did not find any difference between the patients and the controls in WB, the results of IHC showed a greater number of follicular cysts in PCOS compared to healthy controls. They suggested that high aggregation of androgen in PCOS patients' ovaries may be associated with the early steps in androgen biosynthesis (6). Accordingly, seven common *StAR’*s polymorphisms, which are involved in cholesterol binding, were used. Although in 2009 investigators showed an association between polymorphisms in *StAR* gene and the age of menopause (21), and also Terry *et al* in 2010 studied *StAR* polymorphisms in endometrial cancer (22), but for the first time in this study, we used common *StAR* SNPs to find the relationship between these polymorphisms and PCOS. 

Therefore, 45 PCOS patients were compared to 40 normal ovulatory women (male factor) and 40 fertile controls and chosen polymorphisms were detected by PCR-RFLP method. Heterozygous form of SNP rs137852689 (Ala218Val) was observed in all three studied groups, but the result was not significant (p= 0.12). 

Since, this is a pilot study, for enhancing the statistical power, increasing of the study population was recommended. Although previous study emphasized that biosynthesis of high androgen in patients with PCOS occurred at the earliest step of steriodogenesis through *StAR* protein, and increase in the amount of cholesterol resulted in biosynthesis of more androgen in the PCOS ovary (6), but the relationship was not found between these SNPs and PCOS in our study. 

Finally, it seems that changes in amino acid active sites and it’s effect on steroid biosynthesis can associate with the PCOS. Also, the medical records of group 1 group 3 that were investigated in this study, confirmed the results of ESHRE workshop group (23).

## Conclusion

Since, StAR has a critical role in transporting of cholesterol and steroidogenesis, changing in the active site of this gene might affect steroidogenesis and causes hyperandrogenemia which is one of the most important PCOS criteria. So it seems, evaluation of the active amino acids sites should be investigated and also the study population should be increased.

## Note

***This article is extracted from M.Sc. Thesis (Azadeh-Sadat Nazouri) ***
